# Supplementary material for: The genome sequence of the Neglected Rustic moth, Xestia castanea (Esper, 1798) (Lepidoptera: Noctuidae)
Source: Wellcome Open Res. 2026 Apr 17;11:226. [Version 1] doi: 10.12688/wellcomeopenres.26316.1 (PMC13179489; doi:10.12688/wellcomeopenres.26316.1)
Supplement: Peer review history

# Reviewer report: Reviewer response for version 1

**DOI:** 10.21956/wellcomeopenres.28981.r153687

**Published:** 2026-05-15

**Authors:** Shangzhe Zhang

**Affiliations:** 1 Department of Entomology, University of California Riverside (Ringgold ID: 8790), Riverside, California, UK

The authors presented genomic assembly for *Xestia castanea*, which constitutes important input to the ToL project. I do think the protocol and results are rigorous, am just listing a few points that were confusing and can be improved.

 1.  I was very interested in the supernumerary B chromosomes reported, however I could not find any method description on how the authors defined them. In the result section, I don't see plots to enlighten me what are the B chromosomes look like.

 2.  In Figure 6, I believe the axis labels are understandable, but it would be neat to make them clearer to wider group of readers.

 3. The EBP metric is quite a new concept for me, it would be helpful to brief how it works and when to tell the assembly is in good quality in the methods section.

 4. From the aspect of using the genome as a reference in future works, it's good to have Z and W haplotypes being allocated to one haplotype but this needs some clarification.

Are sufficient details of methods and materials provided to allow replication by others?

Yes

Is the rationale for creating the dataset(s) clearly described?

Yes

Are the datasets clearly presented in a useable and accessible format?

Yes

Are the protocols appropriate and is the work technically sound?

Yes

Reviewer Expertise:

Genomics, genomic structural variants, evolutionary biology

I confirm that I have read this submission and believe that I have an appropriate level of expertise to confirm that it is of an acceptable scientific standard.

# Reviewer report: Reviewer response for version 1

**DOI:** 10.21956/wellcomeopenres.28981.r153685

**Published:** 2026-05-08

**Authors:** Vahap Eldem

**Affiliations:** 1 Istanbul University, Istanbul, Turkey

The manuscript presents a chromosome-scale, haplotype-resolved genome assembly for the Neglected Rustic moth, Xestia castanea. The study describes the collection and taxonomic verification of a female specimen from Beinn Eighe, Scotland, followed by PacBio HiFi sequencing, Hi-C scaffolding, mitochondrial genome assembly, manual curation, quality assessment, and gene annotation. The final assembly is reported as two haplotypes with total lengths of 721.78 Mb and 685.53 Mb, with high BUSCO completeness for haplotype 1 and chromosome-level scaffolding for most of the sequence. The manuscript also provides sequencing metadata, assembly statistics, chromosome/pseudomolecule tables, k-mer and BUSCO quality metrics. Data availability is clearly provided through ENA accessions. Overall, the article represents a useful genomic resource for X. castanea and for comparative studies of Noctuidae and Lepidoptera more broadly.

 Several points in the description of the haplotype-resolved assembly would benefit from clarification to ensure consistency between the abstract, tables, and main text.

 The abstract states that haplotype 2 is mostly scaffolded into 32 chromosomal pseudomolecules, and table 2 lists the assembly level of haplotype 2 as chromosomes. However, the assembly statistics section states that haplotype 1 was curated to chromosome level, while haplotype 2 was assembled to scaffold level. This appears inconsistent and may require editing so that the abstract, table 2, and main text describe the status of haplotype 2 consistently.

 The abstract mentions a supernumerary chromosome B2 in haplotype 2, whereas table 3 lists both B2 and B3 under haplotype 2. It would be helpful to clarify whether both B2 and B3 are interpreted as supernumerary chromosomes, B-chromosome-derived scaffolds, or alternative/partial representations of the same supernumerary element.

 Table 2 reports 32 chromosomes for haplotype 2, while Table 3 lists 1A, 1B, chromosomes 2–29, B2, and B3, with no W or Z chromosome. Although this gives 32 named pseudomolecules, the biological interpretation is not fully clear. In particular, whether 1A and 1B represent a split chromosome 1, two independent chromosomal scaffolds, or another structural interpretation may need clarification. The absence of W/Z designations from haplotype 2 may also be explained briefly.

Are sufficient details of methods and materials provided to allow replication by others?

Yes

Is the rationale for creating the dataset(s) clearly described?

Yes

Are the datasets clearly presented in a useable and accessible format?

Yes

Are the protocols appropriate and is the work technically sound?

Yes

Reviewer Expertise:

Genomics, Bioinformatics

I confirm that I have read this submission and believe that I have an appropriate level of expertise to confirm that it is of an acceptable scientific standard.
